# Evaluation of the Adhesion between Aggregate and Asphalt Binder Based on Image Processing Techniques Considering Aggregate Characteristics

**DOI:** 10.3390/ma16145097

**Published:** 2023-07-19

**Authors:** Min Li, Jian Wang, Zibao Guo, Jingchun Chen, Zedong Zhao, Jiaolong Ren

**Affiliations:** 1 School of Civil Engineering and Geomatics, Shandong University of Technology, Zibo 255000, China; 22507020009@stumail.sdut.edu.cn (M.L.); 21507020787@stumail.sdut.edu.cn (J.W.); 21507020774@stumail.sdut.edu.cn (J.C.); 2 CCCC First Highway Northwest Engineering Co., Ltd., Xi’an 710110, China; cdcfllmdqs@163.com; 3 School of Transportation and Vehicle Engineering, Shandong University of Technology, Zibo 255000, China; 20402010140@stumail.sdut.edu.cn

**Keywords:** aggregate–asphalt adhesion, evaluation method, image processing techniques, aggregate characteristics, water boiling test

## Abstract

Aggregate–asphalt adhesion plays an important role in the water stability of asphalt concrete. In various test standards of different countries, it is evaluated via the subjective judgment of testers using the boiling water test. The subjective judgment in the test method is detrimental to the accuracy of the adhesion evaluation. However, there is no quantitative evaluation method for the aggregate–asphalt adhesion in existing studies. Moreover, the effects of aggregate shape on adhesion are also not discussed and stipulated. Hence, an innovative method based on the Chinese boiling water test and image processing technique is put forward to quantificationally evaluate the aggregate–asphalt adhesion. Moreover, the effects of aggregate shapes on adhesion are also investigated via the proposed method from a view of aspect ratio and homogeneity. Results show that the peeling of the asphalt membrane on the aggregate surface is more serious as the complexity of the aggregate shape increases after the boiling water tests, while the effect degree gradually decreases. The effect of aspect ratio on the peeling status of asphalt membrane is lower than that of aggregate homogeneity.

## 1. Introduction

Pavement diseases greatly influence the use quality, passenger comfort, and traffic safety of pavement structures. The inspection of raw materials before pavement construction can significantly control the occurrence of pavement diseases [1]. Potholes are one of the main diseases in asphalt pavement at present, resulting from a lack of water stability of asphalt concrete [2]. The water stability of asphalt concrete mainly depends on the adhesion between aggregates and asphalt binders [3]. In order to ensure the water stability of asphalt concrete, the adhesion between aggregates and asphalt binders is a required inspection in the standards of different countries. Table 1, Table 2 and Table 3 illustrate the adhesion evaluation method, namely the boiling water test, in the Chinese test standard [4] and the ASTM test standard [5], respectively.

As shown in Table 1, Table 2 and Table 3, it can be found that there is only standard practice of the boiling water test in the ASTM test standard, while there is no evaluation method for the adhesion. In the Chinese test standard, although an evaluation method for the adhesion level is provided according to the peeling area of the asphalt membrane on the surface of the aggregates, there is no detailed calculation method for the peeling area. In other words, the adhesion can only be evaluated via the subjective judgment of testers and observers for the peeling area. Obviously, the uncertain impact of subjective factors on the adhesion evaluation is inescapable. In fact, the explanation items of the Chinese test standard point out the reason that there is no evaluation method in the ASTM test standard is due to the effect of subjective factors. It is detrimental to the accuracy of the adhesion evaluation, especially for the criticality of different adhesion levels.

In this case, some new methods are put forward to evaluate the adhesion between aggregates and asphalt binders. Hefer et al. and Bhasin et al. [6,7] put forward a new method, namely the Wihelmy hanging piece method, to determine the adhesion via advance angles and retreat angles of asphalt binder. Hamzah et al. [8] adopted the direct tension test to establish a new method to evaluate the adhesion grade. Shen et al. [9] evaluated the adhesion via tension test, net adsorption test, and scanning electron microscope. Liu et al. [10] proposed an improved evaluation method for adhesion via measuring the thickness of asphalt membrane based on the contact angle test, scanning electron microscopy, and energy spectrum analysis. Ingrassia et al. [11] investigated the adhesion properties between aggregate substrates and the binder composed of reclaimed wood bio-oils and bio-adhesives after different aging degrees based on the asphalt bond strength (BBS) tests. D’Angelo et al. [12] adopted the same method to analyze the adhesion characteristics between plastomeric binder blends and aggregates. Liu et al. [13] judged the adhesion level based on the PosiTestAT-A adhesion test. Ji et al. [14] and Anastasiya et al. [15,16] evaluated the effect of bio-oil on asphalt adhesion using the sessile drop method from a view of surface free energy of asphalt, bio-oil, and aggregates. In addition, Anastasiya et al. [17,18] also studied the adhesive properties of the bio-oil/asphalt blend via a probe tack test and lap shear test. Although these methods could provide quantitative evaluation methodologies for adhesion inspection, the adopted test methods were not common for engineering applications. It was not beneficial to the engineering application.

Subsequently, with the development of computer science [17,18,19,20], image-processing techniques were tried to investigate the adhesion between aggregates and asphalt binders. Park et al. [21] and Nazirizad et al. [22] analyzed the effect of anti-stripping additives on the water stability of asphalt concrete based on the standard measurement method (water boiling water test) and image processing technique. They considered that the image processing techniques could obtain better efficacy than unaided viewing when evaluating the adhesion. However, although these studies proved that the image processing techniques had the potential to improve the evaluation method for the adhesion, they mainly focused on investigating the efficiency of different anti-stripping additives and did not essentially optimize the current evaluation methods. The issues of the evaluation method for the adhesion in current standards have not been solved.

In addition, the characteristics of different aggregates have significant differences, even for the aggregates with the same specification. It inevitably affects the adhesion evaluation. However, only Shen et al. [9] and Ji et al. [23] investigated the effect of microscopic structures and water-absorption characteristics of aggregates on the adhesion, respectively. Unfortunately, the effects of aggregate shape on adhesion have not been discussed and stipulated in existing studies and standards.

Hence, the objective of this study contains the following two points:

- Establish an evaluation method for the adhesion to reduce the impact of subjective factors considering the usability and popularization;
- Investigate the effect of aggregate shape on the adhesion to further improve the proposed evaluation method.

Based on this, an innovative method based on the Chinese boiling water test and image processing technique is put forward to quantificationally evaluate the adhesion between aggregate and asphalt binder. Moreover, the effects of aggregate shapes on adhesion are also investigated via the proposed method from a view of aspect ratio and homogeneity.

## 2. Materials

In this study, the #70 base asphalt binder and basalt aggregates are adopted to implement the adhesion experiments. Their technical parameters are listed in Table 4 and Table 5, respectively.

## 3. Evaluation Method of the Adhesion Based on the Image Processing Technology

In this study, the evaluation method of the adhesion contains two parts: lab experiment and its result analysis. Considering the usability and popularization, the lab experiment is optimized and implemented based on the Chinese boiling water test. Moreover, according to the existing studies, an image processing technique is adopted to treat the experiment results to evaluate the adhesion.

### 3.1. Lab Experiments (The Chinese Water Boiling Test)

**Figure 1:**
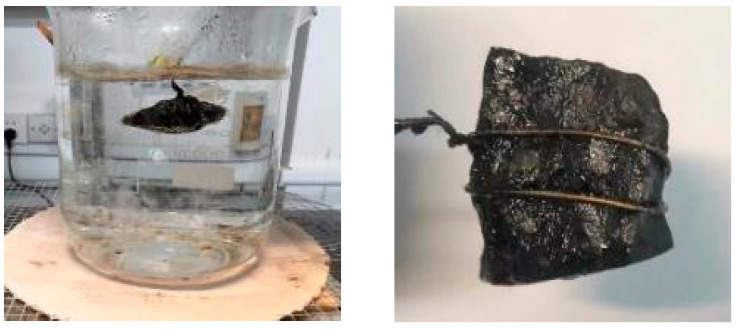
A sketch of the Chinese boiling water test.

In the Chinese test standard, the boiling water test is adopted to judge the adhesion level between aggregates and asphalt binders via the peeling degree of asphalt membrane on the surface of one coarse aggregate in the boiling water, as shown in Figure 1.

**Figure 2:**
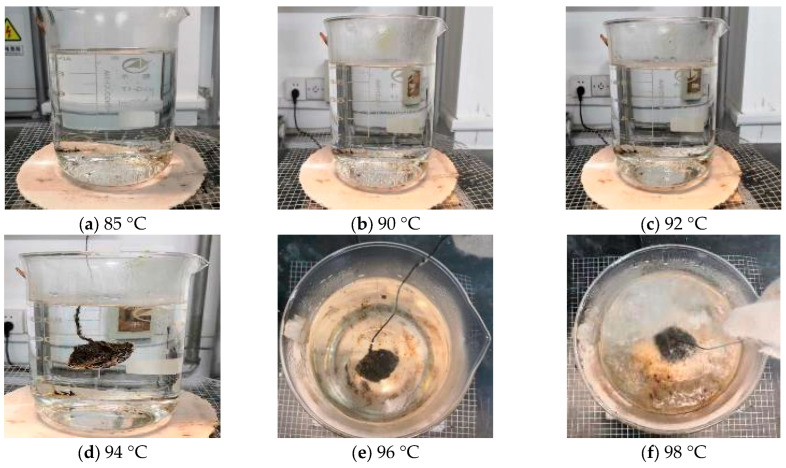
Boiling state at different temperatures.

During the test, the factors affecting the test results are boiling time and boiling temperature. The boiling time has been set at 3 min in the Chinese test standard, while the boiling temperature is not specified. It only describes that the water should be under a micro-boiling state but without generating bubbles. However, the micro-boiling state is difficult to judge, which is detrimental to test standardization. Hence, the boiling water tests at different temperatures (85 °C, 90 °C, 92 °C, 94 °C, 96 °C, and 98 °C) are implemented in this study to determine the boiling temperature, as shown in Figure 2. The rate of heating used in this study is 10 °C/min.

As shown in Figure 2, it can be found that there is no bubble and vapor at 85 °C. When the temperature reaches 90 °C and 92 °C, the vapor can be observed and gradually increases while there are still no bubbles. When the temperature reaches 94 °C, the bubbles begin to generate. Although dissociative asphalt membrane does not appear in the water, many bubbles have been generated on the surface of the aggregate. When the temperature exceeds 96 °C, lots of dissociative asphalt membranes can be observed, accompanied by a large number of bubbles and vapors, which is not permitted in the Chinese boiling water test. Hence, the recommended boiling temperature in this study is 92 ± 1 °C.

### 3.2. Adhesion Evaluation

In this study, the image processing technique based on the software “Image J 1.51j8” [24,25,26] is adopted to obtain the peeling area of the asphalt membrane on the surface of the aggregate. The process of the method is as follows.

**Figure 3:**
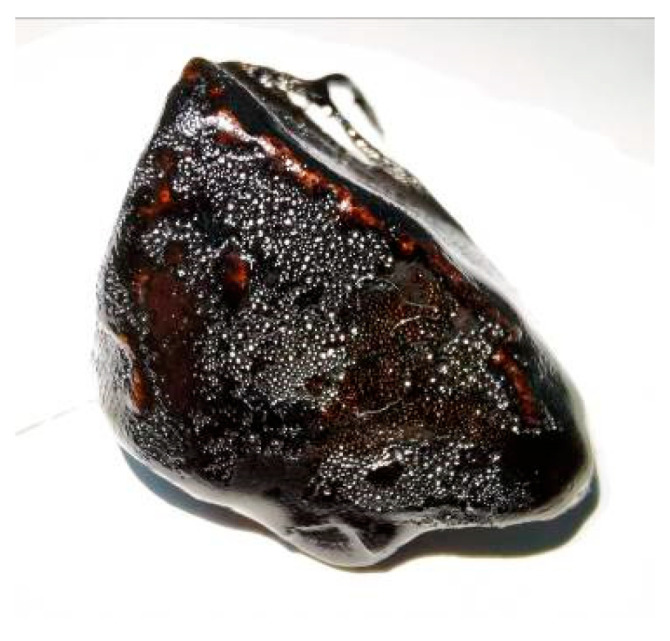
The aggregate after boiling water test on a white slab.

**Figure 4:**
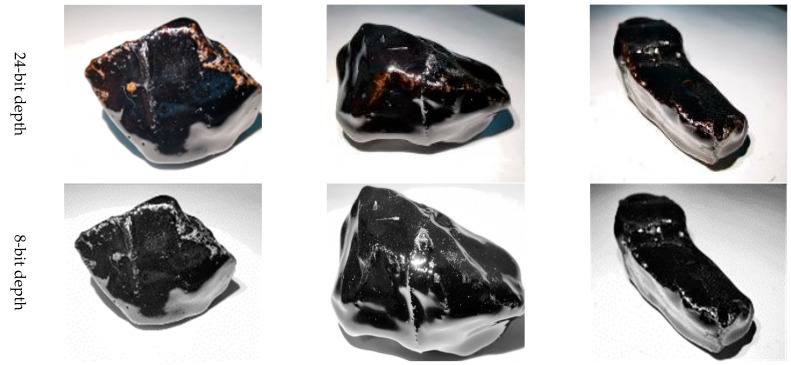
The transformation of the image from 24-bit depth to 8-bit depth.

**Figure 5:**
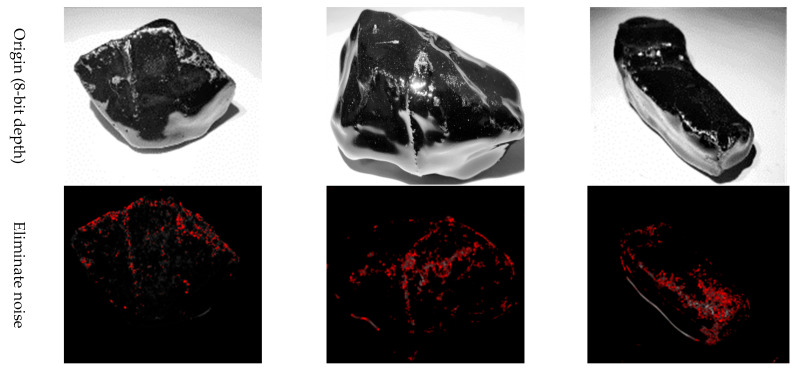
The images after eliminating the background.

**Figure 6:**
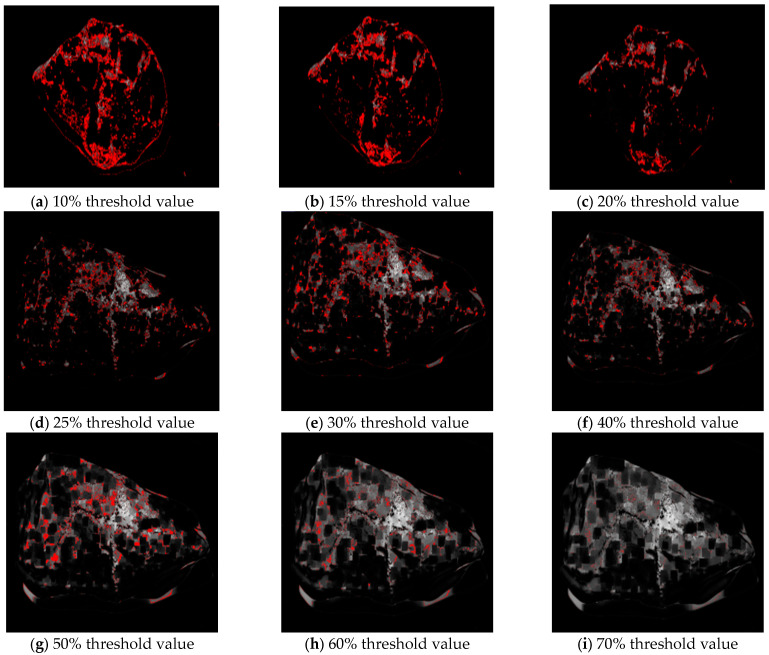
The images after eliminating the background using different threshold values.

- Put the aggregate after the boiling water test on a white slab, as shown in Figure 3. Considering the aggregate is underslung during the boiling water test, there is no peeling zone at the bottom surface of the aggregate owing to the fluidity of the asphalt membrane at high temperatures. Hence, the bottom surface should be in contact with the slab. It should be explained that the soft asphalt binder may not conform to the assumption that there is no peeling zone at the bottom of the aggregate owing to its high fluidity at high temperatures. Hence, one limitation of this study is that the proposed method may not be appropriate for the asphalt binder with a low softening point. However, in fact, soft asphalt is rarely adopted in pavement engineering in China because the softening point of the used asphalt must meet minimum standards.

- Apply a stable light source to illuminate the aggregate. In order to reduce the shadow, it is better to apply the light source from three directions. If the condition is limited, the light source should be in a vertical direction.
- Obtain at least three images of the aggregate via camera equipment from different angles. The image should be larger than 96 dpi.
- Owing to the obtained images using traditional camera equipment, they are usually 24-bit depth and are difficult to be treated using the “Image J”. Hence, these images should be transformed to 8-bit grayscale depth via “Image J” command “Type/8-bit”, as shown in Figure 4. It should be explained that the aggregate is placed on a white paper with a rough texture. The rough texture on the paper can moderate the intensity of light reflection so as to reduce the glare as low as possible. In addition, the color of the area where asphalt peels from aggregate is different from that of the area where the glare occurs. The former is mostly gray and cyan, and the latter is white. They can be distinguished by adjusting the threshold of Image J.
- Eliminate the background of the 8-bit depth image via the “Image J” command “Process/Subtract Background/Rolling ball radius 20% pixels” (Figure 5). The threshold value is very important for the effect of eliminating background. Figure 6 plots the images after eliminating the background using different threshold values.

In Figure 6, the red zone represents the peeling zone of the asphalt membrane. It can be found that the area of the red zone decreases as the threshold value increases [27,28,29,30]. When the threshold value exceeds 30%, the identifiability of the red zone will be reduced largely. However, it does not mean that the smaller the threshold value is, the better. The sensibility of the red zone increases as the threshold value decreases. When the threshold value is lower than 15%, many useless messages are contained in the red zone. Hence, by comparing the area of the red zone and the real peeling area of the asphalt membrane, the optimal threshold value is selected as 20%.

**Figure 7:**
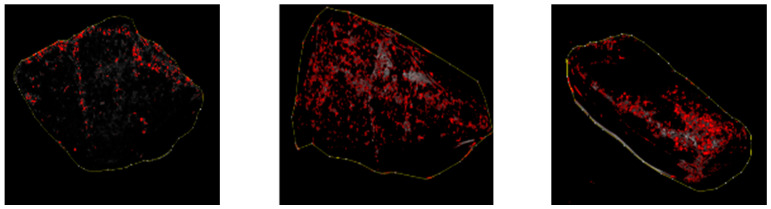
Aggregate outline in the image.

- The aggregate outline can be automatically identified by the “Image J”, as shown in Figure 7.

- Finally, the area of the red zone (peeling zone of the asphalt membrane) and the area of the aggregate can be obtained using the “Image J” command “Analyze/Measure” [31,32,33]. Moreover, the adhesion grade can be quantificationally evaluated according to the peeling area ratio of the aggregate and Table 2. The peeling area ratio RP can be calculated using Equation (1). It should be explained that the above process can be executed in batches.

(1) $$R_{P}=\frac{A_{P}}{A_{A}}\times 100\%$$

where, *A_P_* is the area of the red zone (peeling zone of the asphalt membrane), and *A_A_* is the area of the aggregate.

**Figure 8:**
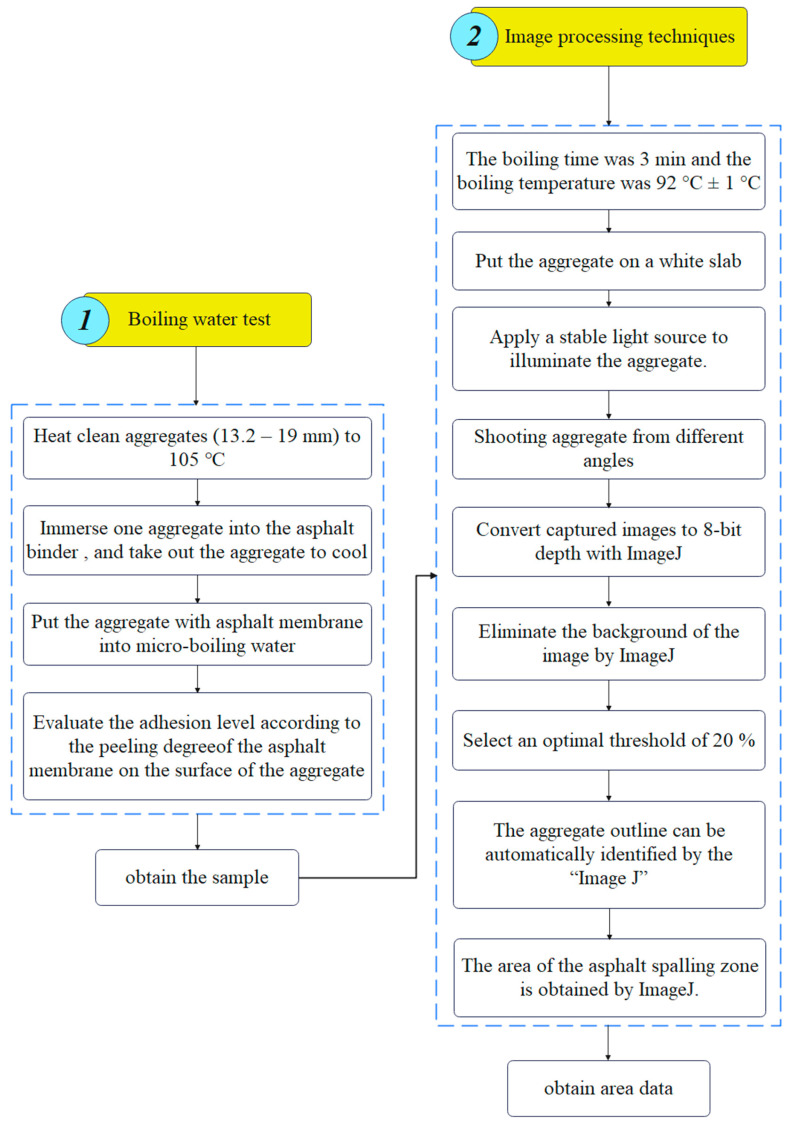
Flow chart of the proposed evaluation method for the adhesion.

The proposed method for the adhesion in this study can be illustrated in Figure 8.

## 4. Effect of Aggregate Characteristics on Adhesion Evaluation

The aggregate shape affects the aggregate–asphalt adhesion; e.g., the asphalt membrane at the sharp zone of the aggregate will be easier to peel during the boiling water test. It also brings some difficulty for the subjective evaluation of the aggregate–asphalt adhesion in traditional methods. Hence, in order to reveal the influence of aggregate shape on the aggregate–asphalt adhesion and explain the feasibility of the proposed method, the aggregates are divided into two categories according to the different homogeneities and aspect ratios. The adhesions of the two types of aggregates are evaluated using the proposed method in this study. The homogeneity and aspect ratio can be characterized using Equations (2) and (3), respectively.

(2) $$H=\frac{\frac{\pi }{6}{\left(\frac{l_{1}+l_{2}}{2}\right)}^{3}}{V}=\frac{\pi \rho {\left(l_{1}+l_{2}\right)}^{3}}{48m}$$

(3) $$A_{t}=\frac{l_{1}}{l_{2}}$$

where, *H* is a self-defined index and is no more less than 1, which represents the homogeneity of the target aggregate. It derives from the Zhang’s study [34]. The lower the *H* is, the more quasi-spherical the aggregate is. *A_t_* is the aspect ratio of the target aggregate and is also no more lessthan 1.0. The larger the *A_t_* is, the more elongated and flaky the aggregate is. *l*_1_, *l*_2_, *V*, *m*, and *ρ* are the longest axis, shortest axis, volume, mass, and density of the target aggregate, respectively.

Some examples of the two types of aggregates are listed in Table 6 and Table 7. Type A (Table 6) represents the aggregates that have similar *A_t_* and different *H*. It can be adopted to reveal the effect of aggregate homogeneity on the aggregate–asphalt adhesion in this study. Type B (Table 7) represents the aggregates that have similar H and different *A_t_*. It can be adopted to reveal the effect of aggregate angularity on the aggregate–asphalt adhesion. Table 6 and Table 7 also present the appearances and general features of different aggregates.

### 4.1. Effect of Homogeneity

Table 8 lists the peeling area ratio and the adhesion grade of the type A aggregates (with different values of *H*) after boiling water tests. The peeling area ratio is obtained according to the proposed method (Section 3.2) in this study. The adhesion grade is obtained according to the traditional method (Table 2) of the Chinese experiment standard. In order to ensure the reliability of the adhesion grade, it is evaluated by a professional team (two professors and one senior experimentalist).

**Figure 9:**
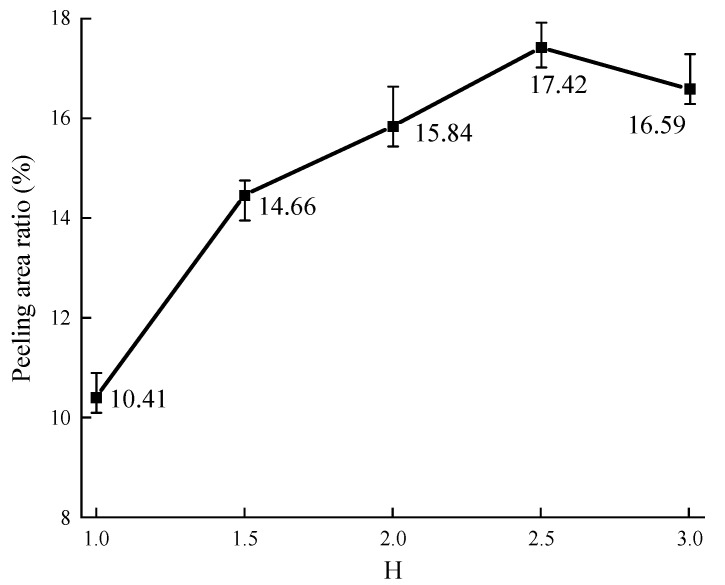
Effect of the value of *H* on the peeling area ratio.

The effect of the values of *H* on the peeling area ratio is shown in Figure 9. The peeling area ratio is calculated as the average value, the same value as *H*.

As shown in Figure 9, it can be found that the peeling area ratio generally increases as the value of *H* increases, especially when the value of *H* is lower than 1.5. Then, a ten-percentage point increase for the value of H makes the peeling area ratio 8.1% increase. When the value of *H* is larger than 1.5, a ten-percentage point increase for the value of *H* only makes the peeling area ratio 1.9% increase. It shows that the increase in aggregate angularity can intensify the peeling of the asphalt membrane while the effect degree decreases as the value of *H* increases. When the value of *H* is lower, the aggregate surface is close to smooth, and the asphalt membrane on the aggregate surface is smooth. After boiling, the peeling of the asphalt membrane is not obvious. When the value of *H* is higher, the edges and corners of the aggregate are more significant, and the asphalt membrane on the aggregate surface is relatively thicker. After boiling, the asphalt membrane easily flows downward and gathers at the pit of the aggregate. As a result, the edges and corners of the aggregate are exposed during the boiling water test. This will influence the evaluation of aggregate–asphalt adhesion grade. Hence, in order to ensure the stability of adhesion evaluation, it is better to select the aggregate with the obvious angularity during the boiling water tests.

In addition, the peeling area ratio slightly decreases when the value of *H* reaches 3.0. It may be due to the experiment errors resulting from a lack of the number of aggregate samples (the shape of aggregates is not common, and the aggregates are not easy to search when the value of *H* exceeds 3.0).

### 4.2. Effect of Aspect Ratio

Table 9 lists the peeling area ratio and the adhesion grade of the type B aggregates (with different values of *A_t_*) after boiling water tests.

**Figure 10:**
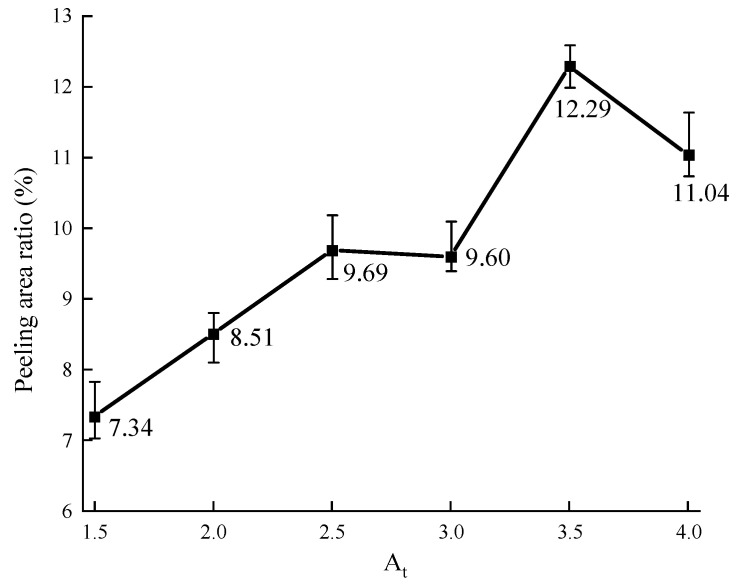
Effect of the value of *A_t_* on the peeling area ratio.

The effect of the values of *A_t_* on the peeling area ratio is shown in Figure 10.

As shown in Figure 10, it can be found that the peeling area ratio generally increases as the value of *A_t_* increases. The peeling of the asphalt membrane at both ends of the aggregate is more serious. However, the effect of the value of *A_t_* on the peeling area ratio is lower than that of the value of *H*. A ten-percentage point increase for the value of *A_t_* on average makes the peeling area ratio 3.4% increased, while on average, 4.5% increased for the value of *H*. It shows that the effect of aggregate flatness on the aggregate–asphalt adhesion is weaker than that of aggregate angularity. Similarly, with Figure 9, the peeling area ratio slightly decreases when the value of *A_t_* reaches 4.0. It also may be due to the experiment errors resulting from a lack of the number of aggregate samples. The shape of aggregates is not common when the value of *A_t_* exceeds 4.0.

In addition, as shown in Table 8 and Table 9 (No. A6, A18, A19, A37, A43, B1, B3, B5, B7, B10, B13, B15, and B18), some adhesion grades obtained by the professional team are different from the results of peeling area ratio obtained by the proposed method of this study. The proposed method is beneficial to evaluate the peeling status of asphalt membrane to accurately judge the adhesion grade.

## 5. Conclusions

In this study, an innovative method based on the Chinese boiling water test and image processing technique is put forward to quantificationally investigate the peeling status of the asphalt membrane. Moreover, two indexes are proposed to characterize the aggregate features (homogeneity and aspect ratio). The effects of aggregate features on the aggregate–asphalt adhesion are analyzed via the proposed method and conventional evaluation method. The proposed method is beneficial for evaluating the peeling status of asphalt membranes to accurately judge the adhesion grade. However, one disadvantage of the proposed method is the high requirement for light sources.

The peeling of the asphalt membrane on the aggregate surface is more serious as the complexity of the aggregate shape increases after the boiling water tests, while the effect degree gradually decreases. When the value of *H* is lower than 1.5, a ten-percentage point increase raises the peeling area ratio by 8.1%. When the value of *H* is larger than 1.5, it only raises the peeling area ratio by 1.9%. In order to ensure the stability of adhesion evaluation, it is better to select the aggregate with the obvious angularity during the boiling water tests. In addition, the effect of aspect ratio on the peeling status of asphalt membrane is lower than that of aggregate homogeneity. A ten-percentage point increase for the value of *A_t_* makes the peeling area ratio 3.4% increased, while 4.5% increased for the value of *H*.

However, the adhesion can be influenced by the chemical interaction between aggregate and asphalt. The chemical interaction depends on asphalt type (e.g., base asphalt with different grades, modified asphalt, etc.) and aggregate lithology (e.g., limestone, basalt, granite, etc.). The effects of these factors on the adhesion will be systematically carried out in our future studies.

## Figures and Tables

**Table 1:** Testing method of the adhesion in the Chinese test standard.

Procedure	Practice
Step 1	Heat clean aggregates (13.2–19 mm) to 105 °C.
Step 2	Immerse one aggregate into the asphalt binder (130–150 °C) for 45 s, and take out the aggregate to cool at room temperature for 15 min.
Step 3	Put the aggregate with asphalt membrane into micro-boiling water for 3 min.
Step 4	Evaluate the adhesion level according to the peeling degree (see Table 2) of the asphalt membrane on the surface of the aggregate.

**Table 2:** Evaluation method of the adhesion in the Chinese test standard.

Adhesion Status	Adhesion Grade
The asphalt membrane is intact.	5
The peeling area is less than 10% of the aggregate superficial area.	4
The peeling area reaches 10–30% of the aggregate superficial area.	3
The peeling area is more than 30% of the aggregate superficial area.	2
The asphalt membrane is completely moved, and the aggregates are bare.	1

**Table 3:** Testing method of the adhesion in the ASTM test standard.

Procedure	Practice
Step 1	Heat 500 mL distilled water to boiling (80–100 °C).
Step 2	Put 250 g of loose asphalt mixture into boiling water for 10 min.
Step 3	Take out the asphalt mixture and obtain the coating rate of asphalt on aggregates.

**Table 4:** Technical parameters of asphalt binder.

Penetration at 25 °C (0.1 mm)	Softening Point (°C)	Ductility (cm)		Viscosity at 60 °C (Pa·s)	After the RTFOT		
		10 °C	15 °C		Residual Penetration Ratio at 25 °C (%)	Residual Ductility at 10 °C (cm)	Mass Loss (%)
67.4	47.4	37.2	>100	31.6	79.3	33.8	0.17

**Table 5:** Technical parameters of aggregates.

Crushed Stone Value (%)	Los Angeles Abrasion Value (%)	Ruggedness (%)	Flat-Elongated Particles Content (%)	<0.075 mm Particle Content (%)	Water Absorption (%)
14.4	16.2	6.2	6.1	0.5	0.81

**Table 6:** Type A aggregates.

No.	*H*	Appearance	Feature
A-I	1.0	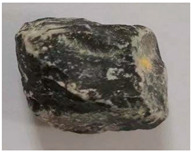	Surface smooth; approximately spherical.
A-II	1.5	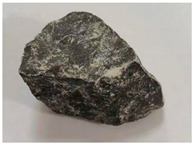	Not obvious angular; polyhedral.
A-III	2.0	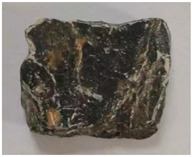	Approximate cube.
A-IV	2.5	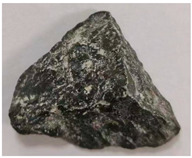	Tetrahedral; obvious angular.
A-V	3.0	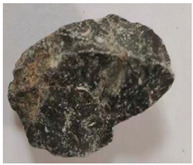	Irregular shape.

**Table 7:** Type B aggregates.

No.	*A_t_*	Appearance	Feature
B-I	1.5	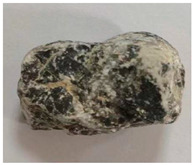	Approximately cylindrical.
B-II	2.0	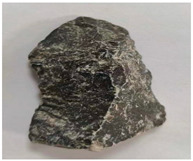	Obvious edges.
B-III	2.5	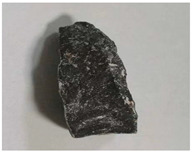	Rectangular shape.
B-IV	3.0	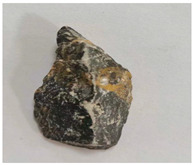	Irregular shape.
B-V	3.5	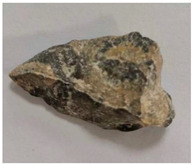	

**Table 8:** Experiment results of type A aggregates.

No.	*H*	Peeling Area Ratio (%)	Adhesion Grade
A1	1.0	9.45	4
A2	1.0	11.25	3
A3	1.5	8.79	4
A4	1.5	8.93	4
A5	1.2	8.06	4
A6	1.2	9.07	3
A7	1.5	10.73	3
A8	1.5	12.77	3
A9	1.5	9.14	4
A10	1.5	9.38	4
A11	2.0	8.04	4
A12	2.0	12.87	3
A13	1.0	10.36	3
A14	1.0	10.60	3
A15	1.5	12.07	3
A16	1.5	12.23	3
A17	2.0	13.46	3
A18	2.0	12.94	4
A19	2.5	11.99	4
A20	2.5	14.36	3
A21	2.0	14.10	3
A22	2.0	15.89	3
A23	1.5	16.13	3
A24	1.5	18.78	3
A25	3.0	18.42	3
A26	3.0	15.58	3
A27	2.5	14.15	3
A28	2.5	18.45	3
A29	2.0	18.54	3
A30	2.0	19.50	3
A31	3.0	15.07	3
A32	3.0	17.27	3
A33	2.0	20.86	2
A34	2.0	18.30	3
A35	2.5	19.38	3
A36	2.5	18.29	3
A37	1.5	18.55	2
A38	1.5	19.28	3
A39	2.0	17.21	3
A40	2.0	18.33	3
A41	1.5	17.32	3
A42	1.5	18.53	3
A43	2.0	19.08	2
A44	2.0	15.23	3
A45	1.5	17.29	3
A46	1.5	17.96	3
A47	1.5	14.82	3
A48	1.5	15.66	3
A49	2.0	15.42	3
A50	2.0	19.68	3
A51	1.5	15.60	3
A52	1.5	15.10	3

**Table 9:** Experiment results of type B aggregates.

No.	*A_t_*	Peeling Area Ratio (%)	Adhesion Grade
B1	2.5	8.38	3
B2	2.5	7.17	4
B3	3.0	9.94	3
B4	3.0	7.71	4
B5	3.0	9.05	3
B6	3.0	11.68	3
B7	4.0	10.16	4
B8	4.0	11.91	3
B9	2.5	12.44	3
B10	2.5	10.75	4
B11	2.0	12.07	3
B12	2.0	10.75	3
B13	3.5	11.47	4
B14	3.5	12.44	3
B15	1.5	11.98	4
B16	1.5	10.70	3
B17	2.0	13.80	3
B18	2.0	13.04	3
B18	3.5	11.85	4
B20	3.5	13.40	3

## Data Availability

The data presented in this study are available in the article.
